# NUB1 and FAT10 Proteins as Potential Novel Biomarkers in Cancer: A Translational Perspective

**DOI:** 10.3390/cells10092176

**Published:** 2021-08-24

**Authors:** Maria Arshad, Nazefah Abdul Hamid, Mun Chiang Chan, Fuad Ismail, Geok Chin Tan, Francesco Pezzella, Ka-Liong Tan

**Affiliations:** 1 Faculty of Medicine & Health Sciences, Universiti Sains Islam Malaysia (USIM), Persiaran Ilmu, Putra Nilai, Nilai 71800, Malaysia; maria@raudah.usim.edu.my (M.A.); nazefah@usim.edu.my (N.A.H.); 2 Department of Molecular Medicine, Faculty of Medicine, University of Malaya, Kuala Lumpur 50603, Malaysia; chanmunchiang@um.edu.my; 3 Department of Radiotherapy & Oncology, Universiti Kebangsaan Malaysia Medical Centre, Jalan Yaacob Latif, Bandar Tun Razak, Kuala Lumpur 56000, Malaysia; fuad2305@yahoo.com; 4 Department of Pathology, Faculty of Medicine, Hospital Canselor Tuanku Muhriz, Universiti Kebangsaan Malaysia, Jalan Yaacob Latif, Bandar Tun Razak, Kuala Lumpur 56000, Malaysia; tangc@ppukm.ukm.edu.my; 5 Tumour Pathology Laboratory, Nuffield Division of Clinical Laboratory Sciences, Radcliffe Department of Medicine, John Radcliffe Hospital, Headington, Oxford OX3 9DU, UK; francesco.pezzella@ndcls.ox.ac.uk

**Keywords:** biomarker, cancer, FAT10, NUB1, NUB1L

## Abstract

Cancer increases the global disease burden substantially, but it remains a challenge to manage it. The search for novel biomarkers is essential for risk assessment, diagnosis, prognosis, prediction of treatment response, and cancer monitoring. This paper examined NEDD8 ultimate buster-1 (NUB1) and F-adjacent transcript 10 (FAT10) proteins as novel biomarkers in cancer. This literature review is based on the search of the electronic database, PubMed. NUB1 is an interferon-inducible protein that mediates apoptotic and anti-proliferative actions in cancer, while FAT10 is a ubiquitin-like modifier that promotes cancer. The upregulated expression of both NUB1 and FAT10 has been observed in various cancers. NUB1 protein binds to FAT10 non-covalently to promote FAT10 degradation. An overexpressed FAT10 stimulates nuclear factor-kappa β, activates the inflammatory pathways, and induces the proliferation of cancer. The FAT10 protein interacts with the mitotic arrest deficient 2 protein, causing chromosomal instability and breast tumourigenesis. FAT10 binds to the proliferating cell nuclear antigen protein and inhibits the DNA damage repair response. In addition, FAT10 involves epithelial–mesenchymal transition, invasion, apoptosis, and multiplication in hepatocellular carcinoma. Our knowledge about them is still limited. There is a need to further develop NUB1 and FAT10 as novel biomarkers.

## 1. Introduction

Identifying biomarkers could aid in clinical decision making since it allows personalised medicine in oncology. There are two major types of biomarkers: *predictive* markers, which direct the use of modified therapies, and *prognostic* markers, which assist the evaluation of malignancy features and clinical trial planning. With the availability of biomarkers, managing cancer therapy has revolved around the tenets of genetics and proteomics. Since then, there is a growing interest in expanding the application of biomarkers in managing cancer patients. Additionally, the clinical potential of monitoring disease using immunohistochemistry is well established, particularly in the analysis of receptor status in breast cancer. However, findings on the use of NEDD8 ultimate buster 1 (NUB1) and F-adjacent transcript 10 (FAT10) proteins as a prognostic biomarker remain scarce. To date, oncologists still find it hard to anticipate the chemotherapy response in many cancer patients in selecting the most efficacious treatment for tumours based on their biology. This literature review is based on the search of the electronic database PubMed.

**Figure 1:**
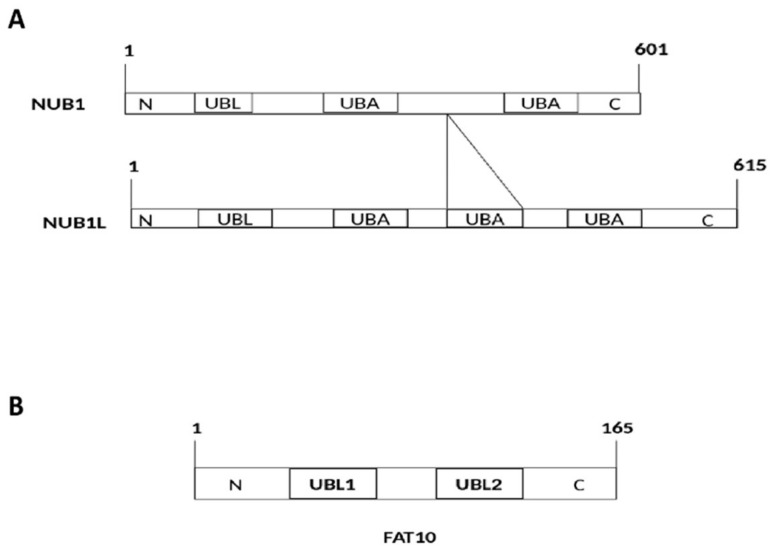
(**A**) Structural and functional domains of NEDD8 ultimate buster-1 (NUB1) and NEDD8 ultimate buster-1 long (NUB1L). UBL: Ubiquitin-like domain (interacts with proteasome); UBA: Ubiquitin associated domain (interacts with ubiquitin-related enzymes). An additional UBA (14 amino acids) domain in NUB1L. (**B**) Structural and functional domains of F-adjacent transcript 10 (FAT10). FAT10 comprises 165 amino acids. UBL: Ubiquitin-like domain (interacts with VWA domain of 26S proteasome).

The NUB1 protein, discovered by Yeh et al. [1], consists of 601 amino acid residues (Figure 1A) with a molecular weight of 69.1 kDa. Its splice variant, the NEDD8 ultimate buster-1 long (NUB1L) protein, interacts with FAT10 protein through non-covalent bonds. Both NUB1 and NUB1L are interferon-inducible proteins that trigger the degradation of neddylation proteins by the ubiquitin–proteasome system [1]. They bind to the proteasome regulatory particle base subunit ribophorin 10 (RPN10) of the 26S proteasome to facilitate the degradation of ubiquitin-like (UBL) proteins, i.e., FAT10 [1].

FAT10 protein is a UBL protein identified in 1996 when sequencing the human major histocompatibility complex gene [2]. It consists of 165 amino acids with a molecular weight of 18 kDa protein, an *N*-terminal domain, and a *C*-terminal ubiquitin-like domain (UBD; Figure 1B). These two domains, with a similarity of 29–36% to ubiquitin, are joined through a hydrophilic linker [2,3]. Both *N*- and *C*-terminal domains show b-grasp protein folds surrounded by b-sheets [4]. In general, UBL proteins require the *C*-terminal to be modifiers [5]. The linker joining the two UBDs of FAT10 then undergoes auto-FAT10ylation through the enzyme UBA6 specific E1 (USE1) [6]. These two UBDs of FAT10 are adequate for effectively mediating proteasomal degradation [5]. FAT10ylation consists of a three-step enzymatic reaction that involves both ubiquitin and FAT10; the UBA6 E1 enzyme for FAT10 conjugation triggers both ubiquitin and FAT10 [7,8]. Although FAT10 has a greater affinity to UBA6 than to ubiquitin, transthiolation and adenylation reactions are slower in FAT10 than the ubiquitination [8,9]. A conjugating E2 enzyme, UBA6-specific enzyme 1 (USE1) has a thioester as an intermediate entity with the FAT10 C-terminus [10]. USE1 goes through self-FAT10ylation in *cis,* mainly at Lys323, to accelerate its proteasomal degradation [11].

With the assistance of proteasome, NUB1L accelerates the degradation of FAT10 degradation up to four times [12]. NUB1L enhances the degradation of FAT10 and their conjugation by recruiting them to the ubiquitin–proteasome system [13]. In addition, the increment of FAT10 expression prognosticates a poorer survival in breast cancer patients [12].

## 2. Interaction of FAT10 and NUB1

Meanwhile, the subunit RPN10 (S5a) of the 26S proteasome serves as an anchoring site for NUB1L, FAT10, and polyubiquitin [5]. Specifically, FAT10 binds to the von Willebrand A (VWA) domain of RPN10 via its *C*-terminal UBL domain and to the UBA domains of NUB1L through its *N*-terminal UBL domain. Consequently, NUB1L interacts with the VWA domain of Rpn10 protein by its *N*-terminal UBL domain. Thus, FAT10 can bind to RPN10 either directly or via NUB1L [5,14].

**Figure 2:**
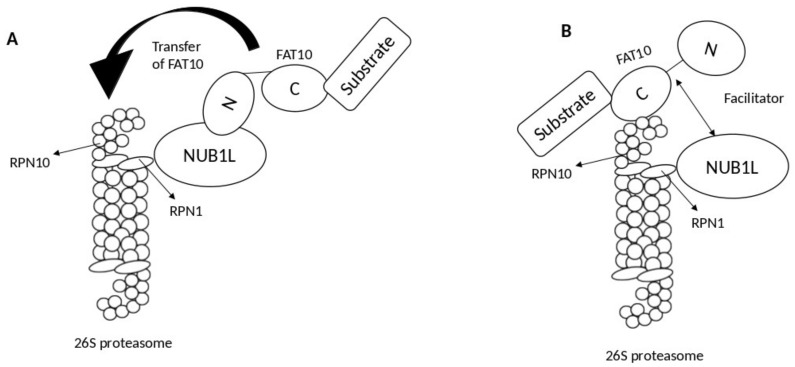
Proposed models show the mechanism of NEDD8 ultimate buster-1 long (NUB1L) in accelerating F-adjacent Transcript 10. (FAT10) degradation by the proteasomal system. (**A**) Model A: the transfer model; NUB1L acts as a receptor and binds to N-terminal of FAT10 and transfer it to ribophorin 10 (RPN10) upon binding to ribophorin 1 (RPN1). (**B**) Model B: the facilitator model; NUB1L acts as a facilitator by binding with RPN1 and later inducing conformational changes within both RPN10 and RPN1 so FAT10 could bind to RPN10, and its degradation can occur.

Two models describe the role of NUB1L as a soluble receptor: the transfer model and the facilitator model. In the transfer model, NUB1L interacts with the *N*-terminal domain of FAT10 and subsequently transfers it to RPN10 upon interacting with ribophorin 1 (RPN1) [14]. By contrast, in the facilitator model, NUB1L, with the 19S regulator of the proteasomal subunit RPN1 (S2), triggers conformational modifications in both RPN1 and RPN10 for interacting with FAT10 and its subsequent conjugation with RPN10 (Figure 2) [14].

The deletion of the UBA domain of NUB1L shows no effect on the degradation of FAT10. However, deleting the UBL domain significantly abolishes the degradation of FAT10. Hence, the binding of the UBL domain of NUB1L to the RPN10 accelerates the degradation of FAT10 by the 26S proteasome [15]. 

## 3. NUB1 Protein Actions in Cancer

NUB1 has been examined in various cancer cell lines, including renal cell carcinoma (RCC), cervical adenocarcinoma, neuroblastoma, rectal adenocarcinoma, and malignant lymphoma [16]. Overexpression of NUB1 in cancer cells is associated with IFN-α induced antimitogenic activities [17]. NUB1 displays anticancer potential in RCC cell lines with S-phase transition and apoptotic properties by cyclin E and p27; p27 inhibits the cyclin E-CDK2 complexes and hence hinders the progression from the G_1_ phase to the S-phase of the cell cycle. Thus, NUB1 protein causes apoptosis and cell-cycle arrest in RCC cells. Overexpression of NUB1 also prevents the proliferation of interferon-α (IFN-α)-resistant RCC cells in vitro [17]. 

Meanwhile, tumour metastasis and invasion are generally characterised by epithelial–mesenchymal transition (EMT). NUB1 protein decreases the expression of EMT proteins, i.e., N-cadherin, matrix metalloproteinase-2 (MMP2), and vimentin, while significantly overexpressing E-cadherin, which is an epithelial marker [18]. This mechanism was identified, and it showed that NUB1 proteins prevent malignancy in gastric cancer cell lines in vitro [18]. 

Another group of proteins related to the malignancy of cancer is the S-phase kinase-associated protein 2 (SKP2) that belongs to the protein complex of Skp, Cullin, and F-box (SCF complex). Deregulating SKP2 or SCF family proteins degrades the p27_Kip1_ in gastric cancer to trigger the malignancy [18,19]. Since NUB1 controls the cell cycle progression via the upregulation of p27_Kip1_ [18], overexpression of NUB1 and NUB1L may prevent the activities of SCF family proteins via suppressing the ligase activity of SCF complexes to treat malignant phenotypes in cancer cells [20]. 

Additionally, the neural precursor cell expressed developmentally downregulates protein 8 (NEDD8), a UBL protein that controls many essential biological functions in the regulation of cell cycles [21]. Although the neddylation of proteins in human cells is inevitable, overexpression of NEDD8 may trigger abnormal effects in cells, leading to the development of cancer [22]. To date, NUB1 and NUB1L have been shown to interact with NEDD8 protein to initiate its degradation through the proteasomal system [22,23]. Specifically, NUB1/NUB1L non-covalently binds to NEDD8 through their UBA domains to prevent its aberrant effects in cells and, ultimately, the development of cancer [22]. 

## 4. FAT10 Protein Actions in Cancer

As a UBL, FAT10 protein regulates cell survival and cell growth. The mRNA and protein of FAT10 are overexpressed in tumours [24]. FAT10 also governs the cellular immune system, and it occurs abundantly in tissues, such as the spleen, lymph nodes, and thymus in humans [25]. The expression of FAT10 is upregulated through the actions of proinflammatory cytokines, i.e., tumour necrosis factor-a (TNF-a) and interferon g (IFNg). An in vivo study showed that IFNγ and TNF-a were responsible for upregulating the mRNA expression of FAT10 in hepatocytes of alcoholic cancer patients [26]. Meanwhile, the degradation of FAT10 could occur only through the 26S proteasome [26]. The upregulated expression of FAT10 accelerates the proliferation and progression of hepatocellular carcinoma, bladder cancer, colon cancer, and cervical cancer (Table 1) [27,28].

It is generally thought that the glucose-regulated protein 78 (GRP78) could phosphorylate and stimulate the P13K/AKT signalling pathway to promote cancer growth [29,40]. The GRP78 protein increases the FAT10 protein expression via the NF-κB pathway, while the FAT10 gene reduces the activity of the tumour-suppressor gene, p53. Thus, the GRP70-NF-κB-FAT10 axis could be a therapeutic target to treat hepatocellular carcinoma (HCC), and further proliferation of HCC cells could be inhibited or reduced [32]. FAT10 protein activates the NF-κB signalling pathway to boost the proliferation of cancer cells [41,42], and in turn, the NF-κB signalling pathway upregulates the pathogenicity and proliferation of HCC [43,44]. 

Furthermore, FAT10 protein is upregulated in the hepatitis B virus (HBV) in association with the HCC tissues [25,45]. The protein stimulates the signalling pathway of protein kinase B/glycogen-synthase kinase 3 Beta (PKB/GSK3β) linked to invasion, EMT, apoptosis, and proliferation in HCC. This finding further shows that FAT10 could serve as a potential biomarker and prospective target to diagnose and treat HBV-related HCC [46]. 

One of the primary causes of cancer progression is chronic inflammation [47]. TNF-α contributes to cancer development by generating chronic inflammation [48]. TNF-α triggers the expression of the FAT10 gene via the TNF receptor 1, inducing the inflammatory signalling pathway of NF-κB in cancer cell lines. TNF-α is also responsible for disrupting the mitotic phase during the cell cycle. This phenomenon could be prevented through the actions of FAT10 protein that induces the activity of TNF-α during cancer pathogenesis by disrupting the cell cycle, chromosomal instability, and inhibition of apoptosis [41].

**Figure 3:**
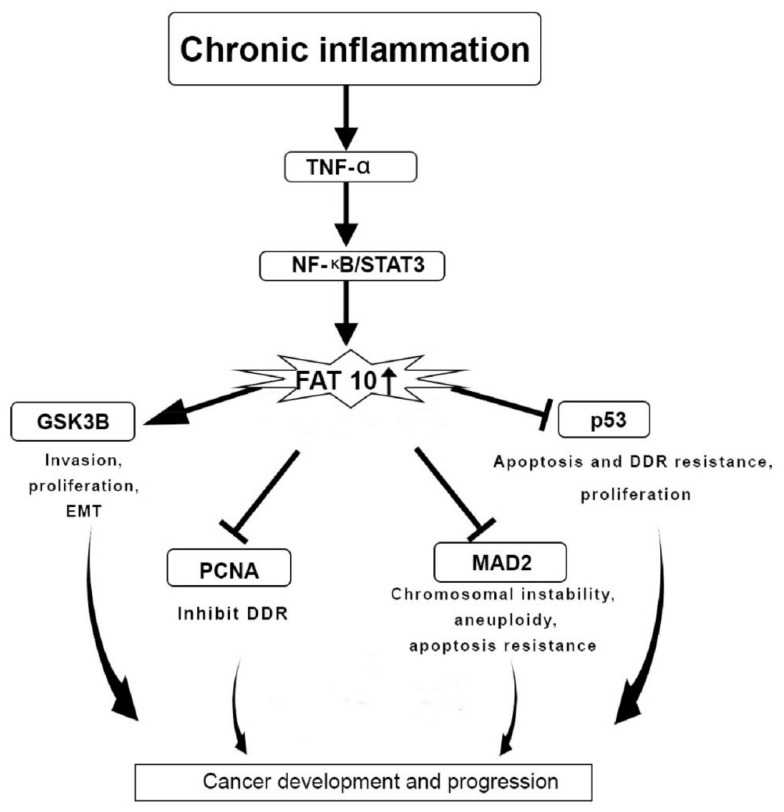
FAT10 protein actions in cancer development and progression. Chronic inflammation regulates the F adjacent transcript 10 (FAT10) gene expression as well as downstream targets through tissue necrosis factor-α (TNF-α) and Nuclear factor- κB/Signal transducer and activator of transcription 3 (NF-κB/STAT3) pathway, which is responsible for upregulating the FAT10 gene expression. Overexpression of FAT10 further mediates the epithelial–mesenchymal transition (EMT), invasion, proliferation, chromosomal instability, DNA damage repair (DDR) resistance, and apoptosis. This leads to cancer development and progression; Glycogen synthase kinase 3 beta (GSK3β); Proliferating cell nuclear antigen (PCNA); mitotic arrest deficient 2 (MAD2); Tumor protein p53 (p53).

The gene expression of FAT10 is linked to the expression of the signal transducer and the activation of the gene transcription 3 (STAT3). The expression of FAT10 is synergistically triggered through the activation of NF-κB (Figure 3) [42]. The STAT3 gene stabilises NF-κB on the promoter region of FAT10 to enhance the expression FAT10 gene [42,49]. The p53 protein is responsible for the degradation of FAT10 protein. Therefore, the interaction of FAT10-p53 is essential to halt cancer progression [50]. There is a transcriptional regulation between STAT3 and NF-κB that impacts FAT10 expression to inhibit p53 expression and hence support cancer and inflammation progression (Figure 3) [41].

In breast cancer, high expression of FAT10 is correlated with a poorer prognosis among patients of breast cancer [30]. Knocking down FAT10 significantly reduces the metastasis potential and EMT abilities of breast cancer cells [30,51]. Additionally, FAT10 protein could bind and stabilise the protein zinc finger E-box-binding homeobox 2 (ZEB2) in breast cancer cells. With the expression of ZEB2, FAT10 protein induces the pro-metastasis effect in breast cancer tissues. Thus, ZEB2 and FAT10 are potential targets to prevent metastasis in the treatment of breast cancer (Table 1) [30].

In HCC, six single-nucleotide polymorphisms have been identified within the 1.3 kb promoter region of the FAT10 gene that is correlated with the overexpression of FAT10 protein in HCC patients [52]. The aberrant methylation is correlated with the FAT10 overexpression in the samples of HCC patients [52]. Thus, the abnormal expression of FAT10 in HCC is likely due to the abnormal methylation pattern of the FAT10 promoter region [52,53]. Genetic modifications at the 5′UTR and variations in the coding region of the FAT10 gene appear to be responsible for inducing HCC in the Chinese Han population [54]. The p53 gene downregulates the expression FAT10 gene during the cell cycle [49,55]. Therefore, dysregulation of FAT10 expression could lead to the development of cancer in p53-defective cells [50]. 

For the two main risk factors of HCC, i.e., alcoholic steatohepatitis and non-alcoholic steatohepatitis, the FAT10 protein is upregulated [56]. FAT10 and ubiquitin proteins are downregulated in neoplastic cells in HCC patients, as compared to normal livers [57]. The downregulation could be due to the dysfunctional FAT10ylation pathway; misfolded proteins disrupt the proteasomal pathway, leading to low protein turnover [58]. An in vivo study demonstrated that dendritic cells transduced with adenovirus-FAT10 triggered anticancer immune reaction against HCC [59].

In bladder cancer, the FAT10 protein promotes the proliferation of cancer cells through direct interaction with the Survivin protein; this direct non-covalent interaction inhibits the ubiquitin-mediated degradation [31]. The expression of FAT10 is upregulated in bladder cancer and it is associated with a poorer prognosis [31]. Together, FAT10 triggers the proliferation of bladder cancer cells by upregulating and stabilising the Survivin protein [31]. 

In glioma, the upregulated FAT10 induces cell invasion, migration, and proliferation of cancer cells, while overexpressed FAT10 stimulates the development of glioma cells in vivo [37,60]. Additionally, overexpression of FAT10 exerts a poorer prognosis among osteosarcoma patients. Overexpressed FAT10 increases invasive and migratory functions in osteosarcoma cells. In HCC, FAT10 protein increases the expression of class I homeobox B9 at both protein and mRNA by suppressing the ubiquitination of b-catenin while boosting the transcription rate of the T cell factor-4 signalling pathway in HCC [31]. 

In non-small-cell lung cancer (NSCLC), a higher expression of FAT10 protein is responsible for chemoresistance. It enhances multiplication, migration, and invasion of cancer cells (Table 1). FAT10 causes NSCLC malignancy and drug resistance by interacting with the signalling pathway of NFkB [51]. The expression of FAT10 expression is downregulated on the retinoid-induced growth inhibition in oestrogen-positive breast cancer [61]. 

Meanwhile, the ubiquitylation of FAT10 is known to increase the rate of FAT10-mediated proteasomal degradation [62]. However, the modification of substrate protein with a single ubiquitin alone is insufficient to trigger the degradation of the 26S proteasome. Instead, the ubiquitin chains will need to be transferred to or assembled onto the substrate protein [5]; i.e., polyubiquitylation is essential for FAT10 self-degradation [5,62]. Thus, through the polyubiquitylation of many substrates, the NUB1 protein affects several essential biological activities, such as transcriptional activities, subcellular distribution, DNA repair, signal transduction, and autophagy [63,64]. 

Additionally, FAT10 proteins take part in multiple cellular functions, such as cell-cycle regulation, nuclear translocation, and signal transduction [65], in which FAT10 proteins bind to the mitotic arrest deficient 2 (MAD2) protein non-covalently. This interaction aids in spindle assembly at the cell cycle checkpoint during the anaphase to maintain the integrity of microtubule spindles during mitosis [66]. The interaction of the FAT10 protein and its complex with the MAD2 protein causes chromosomal instability and the development of malignancy (Figure 3). In B-cell non-Hodgkin lymphomas, the FAT10 protein stimulates cell division and differentiation of dendritic cells and plasma B cells [66]. In general, FAT10 proteins cause genomic instability [67] and regulate the cell cycle, thus promoting the progression of tumour [55]. In contrast, abolishing the interface of FAT10 with MAD2 proteins inhibits the progression of tumour [3]. 

FAT10 protein is overexpressed in various malignancies, such as gynaecological tumours, HCC, gastric tumours, and colorectal tumours (Table 1) [25,50]. The protein contributes to DNA damage response (DDR); dysregulated DNA damage repair at the checkpoints of the cell cycle promotes tumourigenesis [68]. Additionally, FAT10 is located along with the proliferating cell nuclear antigen (PCNA) within the nuclear foci. The DDR-induced FAT10ylation could trigger cellular PCNA degradation [33]. Together, FAT10 and NUB1 could serve as novel prognostic and diagnostic biomarkers to prognosis and predict survivability in cancer patients. 

## 5. Use of NUB1 and FAT10 as Biomarkers in a Clinical Setting

Several studies investigated the correlation between the NUB1 and FAT10 proteins and the survival probability among cancer patients. Table 2 summarises the findings that identified NUB1 and FAT10 as prognostic and predictive biomarkers. Although the concentration of NUB1 mRNA is higher in cancer cells, the depletion of NUB1 protein could lead to G_0_/G_1_ cell cycle arrest in vitro. The knockdown of NUB1 prevents the growth of MDA-MB-231 cell lines in vitro. The cell cycle arrests lead to the death of the breast cancer cells because of the accumulation of p21/p27 proteins in NUB1-depleted cells [69]. 

However, there are contradicting speculations. NUB1 and FAT10 mRNA expressions were overexpressed in numerous cancers relative to adjacent normal cells. Only overexpressed FAT10 was associated with the poor outcome. Meanwhile, the reduced NUB1 protein levels were correlated to higher metastatic prevalence with poorer outcomes [15,20]. The aggressive tumour was characterised by low cytoplasmic NUB1, showing high levels of apoptosis with high proliferation [69]. To date, there have been no studies explaining how a low NUB1 protein level could cause metastasis. It is possible that NUB1 is oncogenic and is associated with less aggressive cancer pathways. NUB1-positive and -negative tumours could be very different in in biological features.

The depletion of NUB1 deactivates ubiquitin E3 ligase pathways, causing cell cycle arrest. However, breast cancer patients with lower survival probability had low cytoplasmic NUB1 protein [69]. Due to this contradicting phenomenon, the mechanism requires further exploration. It is possible that NUB1 behaves differently upon different stressors i.e., hypoxia. Therefore, low cytoplasmic NUB1 level can be an indication of either its low expression or degraded NUB1 protein.

A study found that decreased NUB1 levels were positively correlated to the poorer prognosis of gastric cancer (GC) patients [18]. In contrast, upregulated NUB1 expression prevented cancer cell multiplication and hindered the G_1_/S cell cycle phase transition in GC cells [18]. In the same study, upregulated expression of NUB1 prevented GC cells invasion and migration through downregulating main EMT markers, i.e., vimentin, *N*-cadherin, and by decreasing the expression of matrix metalloproteinases). These results show the importance of NUB1 as a prognostic marker for GC patients [18].

The NUB1 protein ubiquitinates and degrades various substrates, such as p53, cyclin E, and p27_Kip1_ [35,70] by serving as a negative regulator of NEDD8. The tumour-suppressor gene, p27_Kip1_ prevents cyclin E-CDK2 complex from G_1_ to S-phase transition GC cells [18]. Meanwhile, overexpression of FAT10 distinctively prognosticates poorer survival in pancreatic ductal adenocarcinoma (PDAC) patients [71]. The expression of FAT10 is a distinct prognostic factor of survival rate in PDAC patients, which could also serve as a significant diagnostic and therapeutic target for treating PDAC [71]. FAT10 is overexpressed in GC cell lines with the expression of mutated p53 protein, metastasis of lymph node, tumour invasion, and progression. This overexpression suggests that FAT10 is a potential prognostic biomarker for GC in humans and a target to treat cancer [72]. In addition, overexpression of FAT10 is a significant prognostic factor for a poorer survival rate in breast cancer patients with an oncogenic role in the cancer progression [70]. 

## 6. Future Perspectives

NUB1 and FAT10 proteins are important novel determinants in cancer-promoting pathways. Identifying these prognostic biomarkers in metastatic patients might provide insights for new therapeutic targets and predictive elements. At present, studies using NUB1 and FAT10 as biomarkers are still in their early state and mostly limited to the prognostic purpose. There is a need to investigate the predictive aspects of both proteins. Predictive biomarkers could steer tailored therapies for patients based on our enhanced knowledge of the biological behaviour of malignancies. The identified predictive biomarkers could then serve as the cornerstone for developing new anticancer biologicals. 

Most studies were based on retrospective or backward-looking data with insufficient sample size and limited evidence. Further studies with a larger sample size are needed to validate the use of both biomarkers and their association with the distant metastasis of tumour. 

Additionally, evaluating the quality of antibodies for both NUB1 and FAT10 in the market is essential. It is important to standardise the immunohistochemistry protocols for comparative studies. A fully automated staining method with an affordable price should be available for oncologists.

Future studies would need to explore the potential substrates of FAT10 and relate the degradation of substrates to NUB1L’s interaction. Ultimately, the interaction of FAT10 and NUB1L is the foundation upon which cancer inhibition could be based. 

## 7. Conclusions

FAT10 protein is a major player in cancer invasion, metastasis, and development, while NUB1 has a potential anticancer role. An overexpressed FAT10 protein could potentially serve as a biomarker for cancer progression. In contrast, the depleted NUB1 might provide a prognosis for poor survival in cancer patients.

## Figures and Tables

**Table 1:** The actions of FAT10 protein and its involvement with target proteins and pathway in various cancer types; Hepatocellular carcinoma (HCC); 78-kDa glucose-regulated protein (GRP78); Nuclear factor-κB (NF-κB); zinc finger E-box binding protein 2 (ZEB2); mitotic arrest deficient 2 (MAD2); Proliferating cell nuclear antigen (PCNA); SMAD Family Member 2 (SMAD2); Non-small cell lung cancer (NSCLC).

Cancer Type	Remarks	References
Hepatocellular carcinoma (HCC)	GRP78 protein increases FAT10 protein expression via direct activation on the NF-κB pathway.	[29]
Breast cancer	FAT10 protein induces pro-metastasis effect with the help of ZEB2 overexpression.	[30]
Bladder cancer	FAT10 protein non-covalently binds to Survivin protein to inhibit ubiquitin-mediated degradation.	[31]
B-cell non-Hodgkin lymphomas	FAT10 protein non-covalently binds to MAD2 protein to maintain mitosis.	[32]
Colorectal cancer, HCC, Gastric cancer	FAT10 protein disrupts the DNA damage repair response via modification of PCNA protein.	[33,34,35]
NSCLC	FAT10 causes NSCLS malignancy via interaction with NF-κB signalling pathway.	[36]
Glioma	FAT10 protein increases phosphorylation of SMAD2 protein, which triggers FAT10 induced oncogenic activities.	[37]
Neuroblastoma	FAT10 protein stabilises the survivin protein via non-covalent binding.	[38,39]

**Table 2:** Summary of translational NEDD8 ultimate buster 1 (NUB1) and F-adjacent transcript 10 (FAT10) studies that examine the correlation of protein expression to the survival probability of cancer patients. NUB1 and FAT10 protein statuses are identified as prognostic and potentially predictive biomarkers; NEDD8 ultimate buster 1 long (NUB1L); Non-small-cell lung cancer (NSCLC).

Type	Human Sample Types	Sample Size	Antibody Clone and Host Species	Method of Detection and Biomarker Type	Findings	References
Anti-NUB1 and -NUB1L	Gastric cancer patients	116	Ab38438 (Rabbit polyclonal)	Immunohistochemistry/ Prognostic	Reduced NUB1 level associated to poor prognosis of gastric cancer. *p* < 0.05; HR: 0.33 (0.20–0.54)	[18]
	Breast cancer patients	114	4H2 (Mouse Monoclonal Antibody)	Immunohistochemistry/ Prognostic	Low cytoplasmic NUB1 protein level exerts poorer overall survival. *p* = 0.048, HR: 1.779 (1.006–3.346)	[69]
Anti-FAT10 antibody	Bladder cancer samples	133	MBS4750652 (Rabbit Polyclonal Antibody)	Immunohistochemistry/ Prognostic	Higher FAT10 expression in bladder cancer tissues had poorer survival than those with lower FAT10 expression. *p* = 0.002; HR:?	[31]
	Non-small cell lung carcinoma (NSCLC) samples	45	sc-133199 (mouse monoclonal antibody)	Immunohistochemistry/ Prognostic	High FAT10 expression confers quick chemoresistance than the lower FAT10 expression group. *p* = 0.001; HR:?	[36]
	Breast cancer tissues	120	ab168680 (Mouse polyclonal antibody)	Immunohistochemistry/ Prognostic	FAT10 overexpression leads to poor prognostic factor for poorer outcomes of patients with breast cancer. *p* < 0.05; HR:1.563 (1.232–2.531)	[30]
